# Lipid Dynamics, Identification, and Expression Patterns of Fatty Acid Synthase Genes in an Endoparasitoid, *Meteorus pulchricornis* (Hymenoptera: Braconidae)

**DOI:** 10.3390/ijms21176228

**Published:** 2020-08-28

**Authors:** Jiao Wang, Li-Wei Shen, Xiao-Rong Xing, Yu-Qi Xie, Yi-Jiangcheng Li, Zhi-Xiang Liu, Jun Wang, Fu-An Wu, Sheng Sheng

**Affiliations:** 1 Jiangsu Key Laboratory of Sericultural Biology and Biotechnology, School of Biotechnology, Jiangsu University of Science and Technology, Zhenjiang 212018, China; 189310018@stu.just.edu.cn (J.W.); 182211802108@stu.just.edu.cn (L.-W.S.); 172211801110@stu.just.edu.cn (X.-R.X.); 182211802210@stu.just.edu.cn (Y.-Q.X.); lyjc19961209@foxmail.com (Y.-J.L.); liuzhixiang1229@foxmail.com (Z.-X.L.); wangjun@just.edu.cn (J.W.); 198700001882@just.edu.cn (F.-A.W.); 2 The Key Laboratory of Silkworm and Mulberry Genetic Improvement, Ministry of Agriculture, Sericultural Research Institute, Chinese Academy of Agricultural Science, Zhenjiang 212018, China

**Keywords:** *Meteorus pulchricornis*, lipid content, fatty acid synthase genes, lipogenesis

## Abstract

In insect parasitoids, fatty acid synthases (FASs) have received less attention and their roles associated with lipogenesis loss are far from clear. *Meteorus pulchricornis* is a solitary endoparasitoid wasp of many larvae of lepidopteran pests. The lipid content during developmental stages of *M. pulchricornis* was measured; it was higher in the larval and pupal stages but declined from six-day-old pupae. Lipid accumulation constantly decreased in the adult stage, even after feeding on honey solutions. To investigate the roles of FASs in lipid synthesis in *M. pulchricornis*, four FAS genes (*MpulFAS1~4*) were identified from the transcriptome database of *M. pulchricornis*. All FAS genes included full-length open reading frames and shared 72–79% similarity with the sequences of *Microplitis demolitor*. qRT-PCR validation showed that all four *FASs* had the highest expression after the adult wasps were fed on honey diets. *MpulFAS1* and *MpulFAS2* reached their expression peaks at the adult stage but *MpulFAS3* and *MpulFAS4* peaked at the larval stage. To further study the function of FASs, dsRNA injection knocked down the expression of four *MpulFASs* and resulted in a significant decline of lipid content at the adult stage in *M. pulchricornis*. Results from this study suggest that *M. pulchricornis* adults cannot accumulate lipid content effectively and FASs may still contribute to lipid synthesis in the adult stage. This broadens the knowledge on the ability of lipid synthesis in parasitoid wasps and provides insight into the roles of FASs in insects with parasitic life-history traits.

## 1. Introduction

Lipid reserves can support a large and long-term energy source for organisms; they play key roles in survival and reproduction. In insects, lipid accumulation can be stimulated and achieved by digesting a diet rich in sugars and other carbohydrates [1,2]. Lipids can take part in numerous physiological processes and play vital roles in insects. The lipid content of many insects has been found to increase in times of poor nutrition, such as prior to enter into diapause, suggesting that lipid reserves play an essential role in their life histories [3,4]. Although energy reserves are conserved metabolic processes among all insect species, some exceptions have been observed. For example, some insect species do not consume any external resources in the adult stage and alternatively rely on nutrients and energy deposits obtained during their immature stages [5,6,7]. In this scenario, some parasitoid hymenopteran wasps have been reported to lack the ability to accumulate energy supplies, especially for lipid synthesis at the adult stage, which is termed as lipogenesis loss [8].

Previous studies have revealed that feeding on sugar-rich foods, such as honey or nectar, does not lead to an increase in adult lipid content in parasitic wasps [9,10]. This unique life-history trait, in which the larval wasp completes its development exclusively relying on a single host, is predicted to be the main contributor to the loss of lipogenesis [2]. The physiological mechanism underlying this inability to store excess energy or convert sugars into lipids in parasitoid wasps remains unresolved. To clarify possible factors that contribute to this atypical metabolic process, the types of physiological pathways involved in lipogenesis in parasitoid wasps should be investigated. It is accepted that fatty acid synthesis is the primary synthetic pathway in lipogenesis in organisms [11]. However, the molecular mechanism underlying the absence of lipid synthesis has received less attention.

Fatty acid synthase performs the condensation of acetyl coenzyme A (acetyl-CoA) and malonyl-CoA, using NADPH as a reducing equivalent to produce the 16-carbon saturated fatty acid palmitate [2]. Fatty acid synthase genes (*FASs*) are highly conserved and crucial genes in the fatty acid biosynthetic pathway, especially for the synthesis of palmitic acid, which acts as a precursor for a variety of other lipid types [2]. To date, *FASs* have only been identified in a narrow range of insect species, such as the model insect *Drosophila melanogaster* [12]. In parasitoid wasps, *FASs* have been much less studied. The transcription level of *FASs* in *Nasonia vitripennis* did not increase significantly even after short- and long-term sugar feeding; thus, degradation of *FASs* has been predicted to be associated with the loss of lipogenesis in parasitic lifecycle-based insect species [13]. Although the cascade of genes involved in lipogenesis is complicated, making it difficult to determine which genes are pivotal for the lack of lipogenesis in parasitic wasps, studies on *FASs* may be a promising way to uncover this unusual evolutionary trait.

The common cutworm, *Spodoptera litura* (Lepidoptera: Noctuidae) is an important omnivorous insect that causes widespread economic damage to vegetables and other crops [14]. It is considered one of the most destructive insect pests. *Meteorus pulchricornis* (Hymenoptera: Braconidae) is a solitary endoparasitoid wasp of *S. litura* and many other free-living lepidopteran larvae [15]. Extensive studies on *M. pulchricornis* include host selection strategy [16], the effect of sugar feeding on longevity and oviposition performance, patch foraging behavior [15,16,17,18], and the mechanism of immune suppression on host insects [19,20]. Recently, Sheng et al. [9] demonstrated that the lipid content at the life-endpoint was significantly lower than at emergence whenever any kind of sugar diet was supplied. This demonstrated that *M. pulchricornis* was likely to lose the capacity for *de novo* lipid synthesis, or that at least that they cannot accumulate lipids during the adult stage. However, the detailed dynamics of lipid contents, especially at the scale of whole life stages, are still unknown. Furthermore, the molecular mechanism of lipid decline has yet to be uncovered.

The present study measured the lipid content throughout the developmental stages of *M. pulchricornis* and identified candidate FAS genes from a previously constructed *M. pulchricornis* transcriptome dataset. We also performed a series of tests consisting of real-time quantitative RT-PCR (qRT-PCR) validations and RNA interference to investigate the functions of these FAS genes. We aimed to answer the following questions: (1) What is the lipid content dynamic throughout the life history stages of *M. pulchricornis*; (2) what are the functions of FASs in lipid synthesis or accumulation? The information gained from this study can improve the understanding of the biosynthetic mechanism of lipids in parasitoids.

## 2. Results

### 2.1. Lipid Content at Different Stages

**Figure 1:**
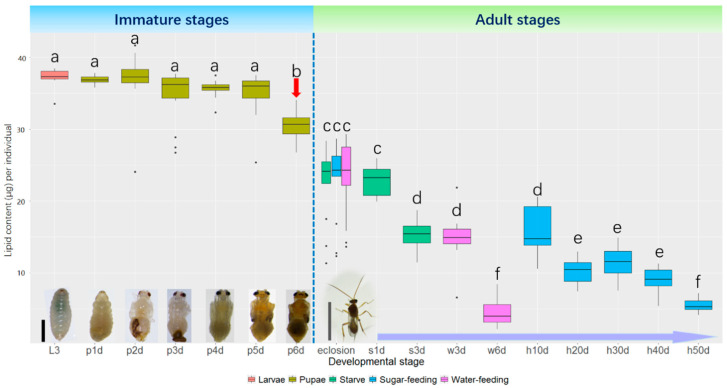
Lipid content at different developmental stages and at different feeding treatments in *Meteorus pulchricornis*. The morphology of each developmental stage and age is illustrated at the bottom of the figure. The scales of the black bar is 2 mm and the grey bar is 5 mm, respectively. L3: third instar larva; p1d~p6d: one- to six-day-old pupae; s1d and s3d: the adult wasps that were undergoing starvation for 1 day and 3 days, respectively; w3d and w6d: the adult wasps that were fed with water for 3 days and 6 days, respectively; h10d~h50d: the adult wasps that were fed with honey solutions for 10 to 50 days. The red arrow indicates the time point that the lipid started to decline significantly. The non-parametric Kruskal–Wallis test was used to detect the significant differences of lipid content among developmental stages and different feeding treatments, followed by pairwise comparisons. The different lowercase letters above the bars indicate significant differences (*p* < 0.05).

The lipid content remained stable from the larval stage to 5-day-old pupae and no significant differences were found among these stages. However, lipid content decreased significantly when the pupae molted into the sixth day. The lipid content at the adult stage was much lower than at the larval or pupal stage. At eclosion, the lipid content was the highest between the adult stages but continuously descended to the endpoint of the adult stage. The lipid accumulation was comparable among the 20-, 30-, and 40-day-old adult female wasps and reached the lowest level at 50 days old. Ten-day-old *M. pulchricornis* had less lipid content than emergency but higher than other ages of adult wasps. When fed on water or deprived of food, the lipid content also decreased compared to that at emergence and reached the lowest level on the third day and sixth day, respectively (Figure 1).

### 2.2. Identification of Fatty Acid Synthase Genes

To examine the correlation between lipid content and FASs, we identified FAS genes from the body transcriptome dataset of *M. pulchricornis*; a total of four FAS genes were obtained (namely *MpulFAS1-MpulFAS4*). All four *MpulFAS*s contained full-length ORFs, ranging in size from 1042 to 2441 amino acid residues (Table S1). The molecular weight of the four predicted proteins ranged from 115.64 kDa to 267.54 kDa and the isoelectric points of the proteins ranged from 5.68 to 6.24. All *MpulFASs* shared 72–79% similarity with *Microplitis demolitor* (Table S1).

### 2.3. Components Phylogenetic Analysis

**Figure 2:**
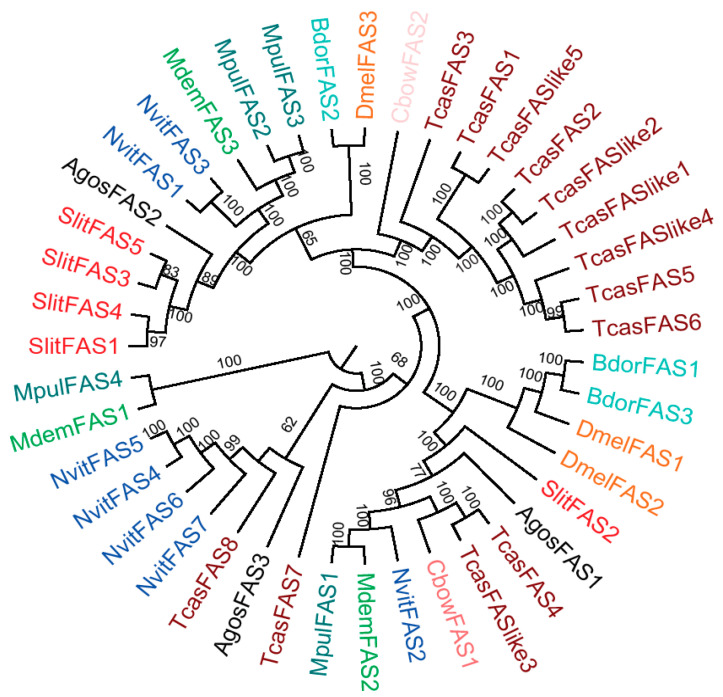
Phylogenetic relationships of FASs amino acid sequences. *Spodoptera litura* (Slit), *Tribolium castaneum* (Tcas), *Microplitis demolitor* (Mdem), *Nasonia vitripennis* (Nvit), *Colaphellus bowringi* (Cbow), *Aphis gossypii* (Agos), *Drosophila melanogaster* (Dmel), *Bactrocera dorsalis* (Bdor), and *Meteorus pulchricornis* (Mpul). The different colors indicate FASs in different insect species. Details of FAS protein sequences are listed in Table S2.

A neighbor-joining (NJ) tree of the FASs was constructed using the protein sequences from *Spodoptera litura*, *Tribolium castaneum*, *Microplitis demolitor*, *Nasonia vitripennis*, *Colaphellus bowringi*, *Aphis gossypii*, *Drosophila melanogaster*, *Bactrocera dorsalis*, and *Meteorus pulchricornis* (Table S2). Four MpulFASs were spread across three sub-branches. MpulFAS2 and MpulFAS3 were clustered into a sub-group and were adjacent to FAS3 in *Microplitis demolitor* (MdemFAS3). MpulFAS1 and MpulFAS4 were clustered with FAS2 (MdemFAS2) and FAS1 (MdemFAS1) into separate sub-branches, respectively (Figure 2).

### 2.4. Expression Patterns of MpulFASs under Different Feeding Conditions

**Figure 3:**
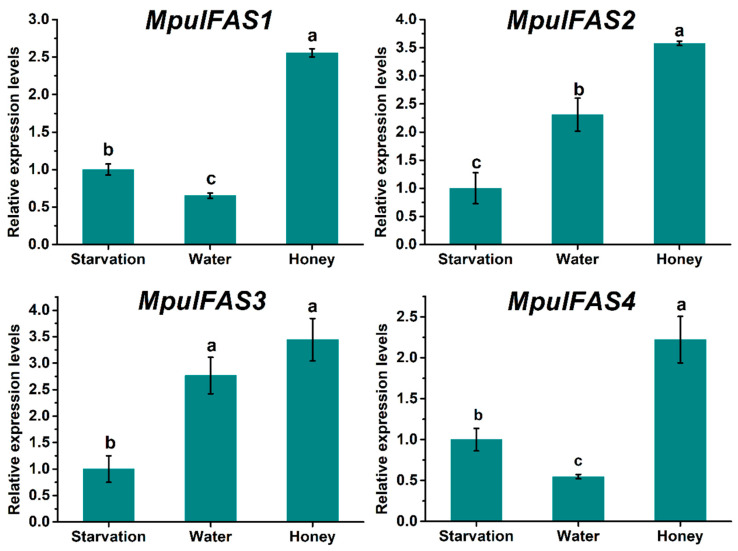
Expression of *MpulFASs* at different feeding treatments in three-day-old adult wasps. The relative expression level was calculated using the 2^−ΔΔCt^ method. A one-way analysis of variance (ANOVA) with Tukey’s post-hoc test was used to check for significant differences in the expression levels of each target gene among the treatments. The different lowercase letters above the bars indicate groups with significant differences (*p* < 0.05).

Expression patterns of *MpulFASs* in three-day-old adult wasps after different diets-feeding were measured. Honey-feeding significantly promoted the expression of four *MpulFAS*s and their expression levels were highest after feeding on honey. *MpulFAS1* and *MpulFAS4* followed a similar expression pattern, in which expression was highest in the honey-fed treatment, followed by starvation and water-fed treatments. The expression levels of *MpulFAS2* and *MpulFAS3* increased in the order of starvation, water, and honey-fed treatments (Figure 3).

### 2.5. Expression Patterns of MpulFASs among Different Life-History Stages

**Figure 4:**
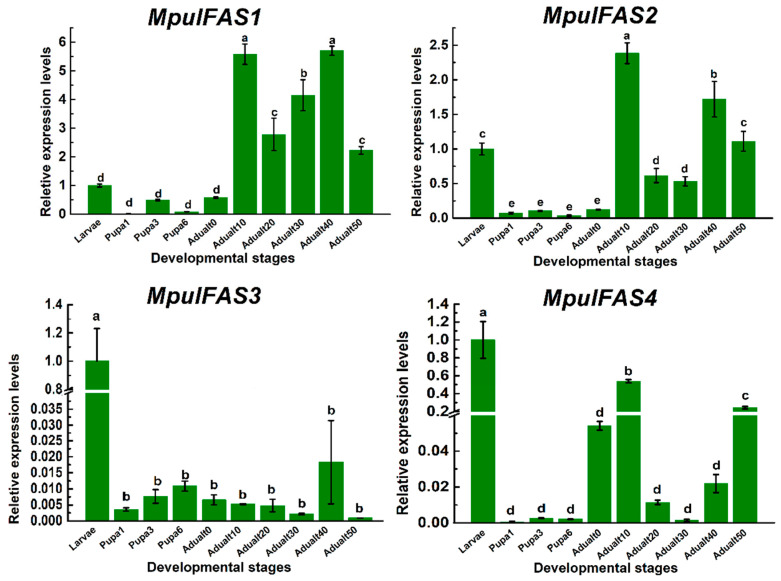
Relative mRNA expression levels of *MpulFASs* at different developmental stages. The relative expression level was calculated using the 2^−ΔΔCt^ method. A one-way analysis of variance (ANOVA) with Tukey’s post-hoc test was used to check for significant differences in the expression levels of each target gene among the treatments. The different lowercase letters above the bars indicate groups with significant differences (*p* < 0.05).

To further clarify the expression patterns of *MpulFAS*s at the adult stage of *M. pulchricornis*, especially for those undergoing honey feeding, we compared the expression levels of *MpulFAS*s at different time points of the adult, larval, and pupal stages. The relative expression levels of four *MpulFASs* varied greatly among life-history stages (Figure 4). The expression levels of *MpulFAS3* and *MpulFAS4* reached their peaks during the larval stages rather than in other life-history stages. In contrast, *MpulFAS1* and *MpulFAS2* were expressed the highest during the adult stage. Interestingly, all four *MpulFASs* were expressed lower in the pupal stages. For the adult stage, the expression levels of *MpulFASs* were significantly different, indicating that the expression patterns of *MpulFASs* at each adult stage were not consistent (Figure 4).

### 2.6. The Analysis of the Function of MpulFASs Using dsRNA

**Figure 5:**
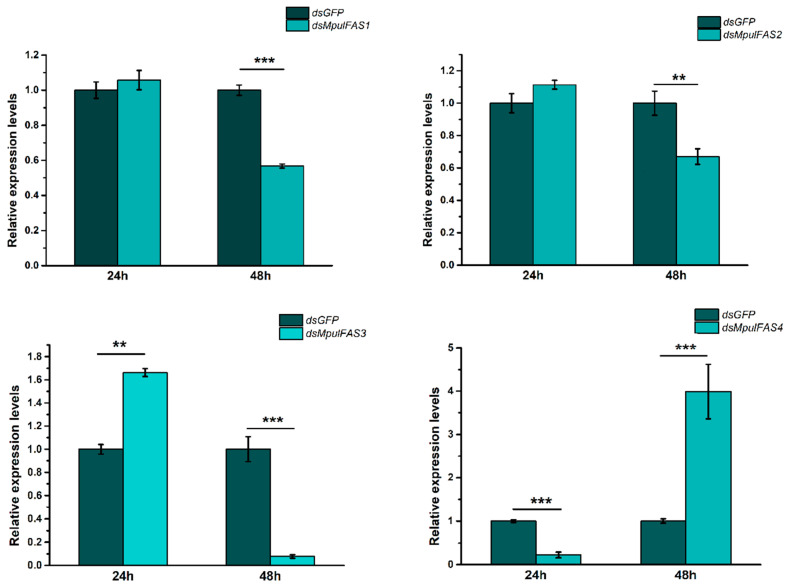
Effect of RNAi treatments of *MpulFAS*s on the expression of 10-old-day adults of *M. pulchricornis.* The relative expression level was calculated using the 2^−ΔΔCt^ method. Differences in the expression levels of each target were compared using a one-way analysis of variance (ANOVA) with Tukey’s post-hoc test. Significant differences are indicated by asterisks (** *p* < 0.01, *** *p* < 0.001).

**Figure 6:**
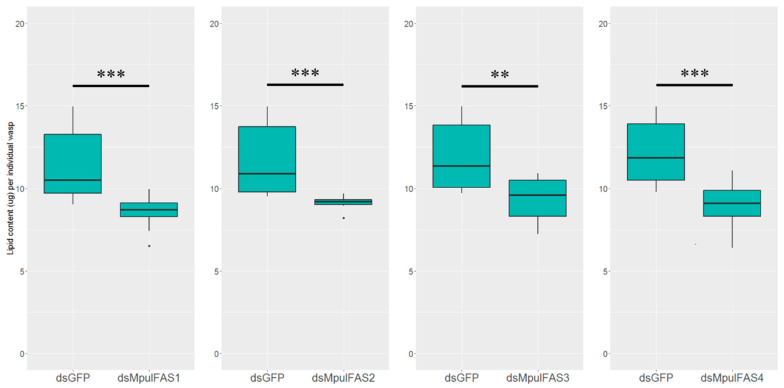
Effect of RNAi treatments of *MpulFAS*s on the lipid content of *M. pulchricornis.* The lipid content was measured at 48 h after knocking down *MpulFAS1*, *MpulFAS2,* and *MpulFAS3*; for *MpulFAS4*, the lipid content was measured at 24 h after dsRNA injection. Differences in lipid content of each treatment were compared using a one-way analysis of variance (ANOVA) with Tukey’s post-hoc test. Significant differences are indicated by asterisks (** *p* < 0.01, *** *p* < 0.001).

In order to investigate the roles of *MpulFASs* in lipid accumulation, *MpulFASs* was silenced by RNAi in 10-day-old adult wasps. The results showed that RNAi treatment decreased the expression levels of four *MpulFASs* in *M. pulchricornis* (Figure 5). The expression of *MpulFAS1*, *MpulFAS2*, and *MpulFAS3* were down-regulated significantly at 48 h after injection, while *MpulFAS4* was down-regulated significantly at 24 h after injection. To further confirm the functions of *MpulFASs*, the phenotypes at the adult stage were assessed for lipid content after silencing *MpulFASs*. The lipid contents all decreased significantly at corresponding test time points (*MpulFAS1-3* at 48 h and *MpulFAS4* at 24 h after injection) (Figure 6).

## 3. Discussion

The association of parasitoid wasp and its host is an excellent model system to study co-evolution between species [21]. During the long-term co-evolutionary process, one side within this association will lose or reduce some traits because of ecological or evolutionary shifts that render the phenotype associated with the trait redundant [13]. The phenotype of lack of lipogenic ability appears to be a common phenomenon in adult parasitoid wasps. Studies have shown that endpoint lipid levels of adult wasps are lower than at emergence, even after intake of a sugar diet [2,22]. For example, the lipid content of adult wasps in *Neochrysocharis formosa* and *Diadegma insulare* continuously declined [10,23]. Similarly, the lipid reserves at the life-endpoint of *M. pulchricornis* females were much lower than those at emergence even after they digested various kinds of sugary foods [9].

However, studies on the lipid content of juvenile parasitoids still receive less attention. Therefore, we prolonged the measurement to immature stages and to a detailed time interval in the adult stage to track the global change in lipid content. The adult stage in *M. pulchricornis* fed on a honey solution, the constant decline of lipid content at each time point demonstrated that: (1) lipid reserves were consumed throughout the adult stage and (2) no further new lipids were recruited despite continuous food digestion, or at least, the synthesis rate of lipids was much lower than the rate at which food was consumed. Similar results for lipid dynamics in wasps fed with water and deprived of food were also detected. Lipid synthesis is usually deemed to be an involuntary dose-dependent physiological process under excessive carbohydrate diets [24]. In contrast, the net increase in lipid reserves depends on the rate of lipid metabolism [22]. It is possible that if lipid content is reduced at a higher rate than it is produced, this could cause the overall decline of lipid levels in parasitoid adult stages [13]. A lack of lipogenesis could be another possible cause. All nutrition of immature parasitoids is derived from a single host and the host can provide sufficient nutrition, including lipids, to their whole life stages, so lipogenesis seems to be an evolutionary redundancy for parasitoid wasps that exhibit special larval life history [25]. This development pattern may result in no extra lipid demand during the adult stages in parasitoids. However, there are two patterns of egg mature in parasitoid wasps, pro-ovigenic, where the female wasps can retain fully mature eggs into the adult stage, or synovigenic, in which only a small amount of mature eggs are produced in the adult stages [26,27,28]. Mature eggs need plenty of lipids in insects [28]; therefore, to test the hypothesis of lipogenesis loss, more parasitoid species that use these two egg maturation types should be tested.

At the larval and pupal stages in *M. pulchricornis*, the lipid content was stable and at a higher level compared to the adult stages. Until now, less attention has been paid to the lipid dynamics at the larval and pupae stages in parasitoids [2]. As pupae, an important and dramatic change in morphology and physiology occurs, where the lipid content was kept at a comparable level, except in 6-day-old pupae, at which point the lipid level began to decrease. Parasitoid pupae inherit sufficient nutrition, including lipid reserves from the larval stage; however, immobility and lack of behavioral activity require less energy investment during this stage. The sudden decline in lipid content in 6-day-old pupae indicated that a lipid decrease starts at the pupal stage [29]. More detailed information about the nutritional change must be obtained on 6-day-old pupae to explain the possible mechanisms.

It has been demonstrated that FASs play a vital role in lipid synthesis in insect species, yet limited FASs have been identified in parasitoid wasps [13,30]. To further explore the cause of lipid dynamics in *M. pulchricornis*, we identified four FAS genes from the *M. pulchricornis* transcriptome dataset [9]. Since lipid synthesis in organisms is a highly conserved function, the number of *FASs* was relatively low among species, including insects. For example, *D. melanogaster* and *Lysiphlebia japonica* have six and three *FASs*, respectively [31], which indicates that *FASs* in different orders of insects are comparable. The phylogenetic analysis showed that FASs of *M. pulchricornis* were adjacent to *M. demolitor*, which also belong to the family Braconidae; therefore, FASs were more conserved within the same family.

Until now, research on the expression patterns of FASs in insects, especially in parasitic wasps, has been scarce. Parasitic wasps can manipulate their hosts to produce sufficient amounts of lipids to meet the needs of developing offspring of parasitoids [32,33,34]; however, this manipulation is restricted to the immature stages, and whether this supply can be maintained at the adult stage is still questionable. In other words, it is unknown whether well-fed adult wasps can abandon the lipid metabolic pathway. Interestingly, all four *MpulFASs* had higher expression in honey-fed groups than in starved or water-fed groups. This result suggested that *FASs* can be activated after feeding on sugary foods, but this was in contrast to the lipid dynamic in three feeding treatments and previous studies regarding *FASs* degradation contributing to the loss of lipogenesis. In *N. vitripennis*, compared to starved groups, the expression of *FASs* in short-term and long-term fed wasps was not significantly changed [13]. Lammers et al. [30] also demonstrated that none of the three *FASs* in *N. vitripennis* (LOC100121447, LOC100122099, and LOC100122083) were unregulated in the sugar-fed treatment. Conversely, *D. melanogaster* accelerated its fatty acid synthesis after sugar-feeding, as indicated by the upregulation of the key gene *fatty acid synthase 1*, suggesting that the expression pattern of *FASs* is variable between Hymenoptera and other insect orders.

To explain the unusual expression patterns of *FASs* under variable feeding conditions, we validated the global expression levels of *FASs* from larval to adult stages with dispersed time points. The results showed that the expression patterns among developmental stages in *M. pulchricornis* varied greatly. The highest expression levels of *MpulFAS1* and *MpulFAS2* were observed in the adult stage, while *MpulFAS3* and *MpulFAS4* had the highest expression at the larval stage. Notably, two *FASs* in *M. pulchricornis* had higher expression in the larval stage than in other stages, which was consistent with lipid dynamics among each developmental stage. Alternatively, another two *FASs* were more highly expressed but with different expression peaks in the adult stage. Specifically, both *MpulFAS1* and *MpulFAS2* had the highest expression after 10 days of honey feeding, where the wasps had a higher lipid content at the adult stage. This divergence in the *FASs* expression pattern revealed functional differentiation; therefore, the explanation for FAS function in fatty acid synthesis is more complicated. Although FASs can be causally linked to lipid synthesis, they also have other important roles in insect physiology. For instance, FASs are various precursors of hormones or enzymes, suggesting that higher transcriptional levels of *FASs* can lead to other physiological functions in the metabolic networks of *M. pulchricornis*.

To further understand the roles of FASs in lipid synthesis in *M. pulchricornis*, we conducted the RNAi experiments to validate the expression levels of *MpulFASs* and the change in lipid contents. Interestingly, after knocking down, all the four *MpulFASs* were down-regulated and eventually resulted in decreased lipid contents in the adult stage. These results strongly suggested that FASs may still make consequences on the lipid synthesis in *M. pulchricornis*. Similarly, the accumulation of lipids decreased significantly in *Colaphellus bowringi* by silencing *FAS*, indicating that FASs play essential roles in lipid synthesis in *C. bowringi* [35].

Metabolic pathway-related lipid synthesis is complicated, and several other genes can play a direct or indirect role in the fatty acid synthesis pathway, such as Acetyl-CoA Carboxylase (ACC) [36,37]. ACC adds a carboxyl group to acetyl-CoA and yields malony-CoA, which is required for fatty acid synthesis. We speculated that, given the lipid content decline in the adult stages of parasitic wasps, one key lipid synthesis-related gene can still be upregulated but other functional genes would be downregulated, and the latter is more important in lipid synthesis than *FASs*. Furthermore, lipid synthesis-related genes in the lipid metabolic pathway are usually antagonistic to each other. For example, high levels of malonyl-CoA can inhibit the activity of carnitine acyltransferase I (CAT1), preventing fatty acid transport to the mitochondria, thereby limiting β-oxidation. Therefore, the upregulation of *FASs* may inhibit other key lipid synthesis-related genes, which contributes to the lipid decrease. Based on this, further studies should be conducted to summarize a global transcriptional change of lipid metabolic genes to screen for candidate genes that are key for lipid lipogenesis loss, in addition to detailed biochemical and molecular validation.

We demonstrated that, during the life history of *M. pulchricornis*, lipid content remains at high levels during the larval and pupal stages but declines from the end of the pupal stage through the adult stage. After honey feeding, the lipid content declined, which confirmed that *M. pulchricornis* adult wasps did not accumulate lipid reserves effectively. *FASs* in *M. pulchricornis* were variably expressed at all life-history stages, and the expression levels of a portion of *FASs* were still high even after saccharide feeding. More importantly, *FASs* may still contribute to lipid synthesis at the adult stage based on RNAi experiments. The present study suggests that the loss of lipid accumulation may be related to other functional genes or more complicated mechanisms.

## 4. Materials and Methods

### 4.1. Insects

Since *M. pulchricornis* is thelytokous, the populations are all l females [38]. Parasitoid wasps were obtained from parasitized *S. litura* larvae collected in soybean fields at the campus of Jiangsu University of Science and Technology in Zhenjiang City, Jiangsu Province, China. Wasps were maintained using L3–L4 *S. litura* larvae as the hosts. Adult wasps were reared in a glass tube (d = 1 cm, h = 8 cm) and provided with a honey solution (10% *w*/*v*) once a day. The host species, *S. litura*, were reared on an artificial diet [39]. Adult moths were fed on a 10% sugar solution and provided with strips of paper as the substrates for egg deposition in organza-covered cages (20 × 20 × 30 cm) [38]. All the parasitoids and host insects were reared in the insectary [26 ± 2 °C, 60–80% relative humidity, and a photoperiod of 14:10 (L:D) h]. For lipid content assays and further molecular experiments, three life stages of *M. pulchricornis* (e.g., larvae, pupae, and adults) were included. Because infant and median instar parasitoid larvae were difficult to collect for biochemical and molecular assays, we chose the final instar (L3) parasitoid larvae to conduct assays.

### 4.2. Lipid Analysis

We compared the lipid dynamics of adult wasps under three feeding conditions: starvation, water-feeding, and honey-feeding treatments. Preliminary tests showed that the adults can live no more than three or seven days without any food supply or only feeding on water, respectively. Therefore, the lipid contents on the first and third day of starved adult wasps and on the third and sixth day of water-fed adult wasps were determined. Then, to illustrate the detailed lipid dynamic, we measured a time series of lipid content at the adult stage, including at emergence, 10, 20, 30, 40, and 50 days old. Meanwhile, the lipid content of larvae (L3) and pupae (from the first day to the sixth day) were determined to compare the global change of lipid dynamics in *M. pulchricornis*.

The lipid content of individual wasps was determined using the vanillin assay [40]. Each individual was crushed with 500 μL chloroform-methanol (*v*/*v* = 1:2) in a 1.5 mL centrifuge tube. After centrifugation at 16,000× *g* for two min, the supernatant was transferred into a new 1.5-mL centrifuge tube. Then, the supernatant was heated at 90 °C for complete evaporation of the solution and 40 μL of 95–98% sulfuric acid was added and heated at 90 °C for 2 min. After that, 960 μL of the vanillin-phosphoric acid reagent was added and left to react at room temperature for 25 min. The solution absorbance at 525 nm was read using a spectrophotometer (Thermo1500, Waltham, MA, USA) and compared to lipid standards for triglyceride [41]. For each life-history stage, twenty individuals were tested.

### 4.3. Identification and Bioinformatics Analysis of FASs in M. pulchricornis

cDNA sequences encoding FASs were retrieved from a previously constructed *M. pulchricornis* transcriptome dataset (GenBank accession number: SRR8981255) [42]. The tBLASTn algorithm (*E*-value < 1 × 10^−5^) was used to identify candidate unigenes encoding putative FASs with the available sequences of these proteins from *Microplitis demolitor*, *Nasonia vitripennis*, *Drosophila melanogaster*, and *Tribolium castaneum*. All candidate genes were manually checked using BlastX against the GenBank non-redundant (nr) protein database at NCBI. The open reading frames (ORFs) of the putative FAS genes were predicted using an ORF finder (https://www.ncbi.nlm.nih.gov/orffinder/). The theoretical isoelectric point (pI) and molecular weight (Mw) were predicted using ExPASy (https://web.expasy.org/compute_pi/). A phylogenetic tree was constructed by MEGA7.0 software using the neighbor-joining method with 1000 bootstrap replications [43].

### 4.4. RNA Isolation and Reverse Transcription

Total RNA samples were extracted using the Trizol reagent (Invitrogen, Carlsbad, CA, USA) following the manufacture’s protocol, and the residual DNA was removed by RNase-Free DNase I (Promega, Madison, WI, USA). Approximately 5 survivors were pooled for preparing one sample. RNA quality and concentration were determined by Nanodrop 2000 spectrometer (Thermo Scientific, Waltham, MA, USA). All RNA samples were reverse transcribed using the PrimeScript RT reagent Kit with gDNA Eraser (Takara, Dalian, China).

### 4.5. qRT-PCR Validation

qRT-PCR was performed using the QuantStudio™ Real-Time PCR system (Applied Biosystems, Foster, CA, USA). The reaction volume was 20 μL, containing 10 μL SYBR Premix Ex Tap II (Takara Biotechnology Co. Ltd., Dalian, China), 0.4 μL each of the forward and reverse primer, 0.4 μL ROX Reference Dye (50×), 1.5 μL cDNA template, and 7.3 μL ddH_2_O. The reaction process was as follows: 5 min at 95 °C, followed by 45 cycles for 15 s at 95 °C, 60 °C for 31 s, and dissociation. A fluorescence melting curve from 55 to 95 °C was used to ensure a single gene-specific peak and the absence of primer-dimer peaks [44]. Each sample was run in triplicate for technical repeats, and three biological replicates were performed simultaneously.

The primers used in the qRT-PCR analysis are listed in Table S3. The *beta-actin* gene was used as the reference gene to normalize the expression of target genes and correct sample-to-sample variation. The primers were designed using the primer designing tool (https://www.ncbi.nlm.nih.gov/tools/primer-blast/) and synthesized by Sangon Biotech (Shanghai, China). Each sample was analyzed in triplicate from three biological replicates, and the relative expression levels of *MpulFAS* genes among the different samples were measured using the 2^−ΔΔCt^ method [45].

### 4.6. The Synthesis of dsRNA Synthesis and Injection

RNA interference (RNAi) was conducted to further analyze the functions of FASs in *M. pulchricornis*. Gene-specific primers for dsRNA synthesis were designed with BLOCK-iT™ RNAi Designer (https://rnaidesigner.thermofisher.com/). According to the manufacturer’s steps, four single-stranded Oligos of each *MpulFAS* were synthesized and are listed in Table S4. The dsRNA was synthesized using an in vitro Transcription T7 Kit (for siRNA Synthesis) (Takara Biotechnology Co. Ltd., Dalian, China) in accordance with the manufacturer’s instructions. The dsRNA of the green fluorescent protein gene was also synthesized as a negative control. The concentration purity of dsRNA was detected by the NanoDrop 2000 spectrophotometer. The dsRNA quality was examined by 2% agarose gel electrophoresis, and the dsRNA subsequently stored at −20 °C until use.

Fifty ng dsRNA of four *MpulFASs* were injected to 10-day-old adults using a Nanoject II microinjector (Drummond Scientific, Broomall, PA, USA), respectively. Fifty wasps were injected for each treatment. Total RNA was extracted after 24 h and 48 h to check the efficiency of RNAi by qRT-PCR. According to the qRT-PCR results, the lipid content per individual wasp was measured based on the time point with the highest interference efficiency and the method was the same as lipid content analysis in Section 4.2.

### 4.7. Data Analyses

Since the assumption of normality was not satisfied, the non-parametric Kruskal–Wallis test was used to detect the significant differences of lipid content among developmental stages, followed by pairwise comparisons using the pgirmess package in R software (version 3.4.0). A one-way analysis of variance (ANOVA) (Systat, Inc., Evanston, IL, USA) with Tukey’s post-hoc test (*p* < 0.05) was used to check for significant differences in the expression levels of each target gene among the treatments. The data analysis was carried out in R 3.4.0 [46].

## Supplementary Materials

Supplementary Materials can be found at https://www.mdpi.com/1422-0067/21/17/6228/s1. Table S1: Sequence information of the identified fatty acid synthetase genes in *M. pulchricornis*. Table S2: Details of FAS protein sequences used for phylogenetic analysis. Table S3: Primers used for real-time qRT-PCR amplification of FASs. Table S4: Primers used to synthesize dsRNA.
