# Supplementary material for: Lipid Dynamics, Identification, and Expression Patterns of Fatty Acid Synthase Genes in an Endoparasitoid, Meteorus pulchricornis (Hymenoptera: Braconidae)
Source: Int J Mol Sci. 2020 Aug 28;21(17):6228. doi: 10.3390/ijms21176228 (PMC7503466; doi:10.3390/ijms21176228)
Supplement: Supplementary file 1 [file ijms-21-06228-s001.pdf]

Table S1. Sequence information of the identified fatty acid synthetase genes in *M. pulchricornis*

Gene name	Acc. No.	ORF full	ORF (aa)	pI	Mw (kDa)	BLASTX best hit				
						Species	Gene description	Acc. no.	E-value	Identity (%)
MpulFAS1	MT680623	Yes	2441	5.68	267.54	*Microplitis demolitor*	PREDICTED: fatty acid synthase	XP_008555885.1	0	79
MpulFAS2	MT680624	Yes	2409	6.24	266.08	*Microplitis demolitor*	PREDICTED: fatty acid synthase	XP_014296122.1	0	73
MpulFAS3	MT680625	Yes	1042	6.04	115.65	*Microplitis demolitor*	PREDICTED: fatty acid synthase	XP_014296122.1	0	72
MpulFAS4	MT680626	Yes	1623	5.92	185.71	*Microplitis demolitor*	fatty acid synthase	XP_014296122.1	0	76

Table S2 Details of FAS protein sequences used for phylogenetic analysis

Name of FAS	Accession Number	Name of FAS	Accession Number
SlitFAS1	XP_022831505.1	NvitFAS1	XP_008203901.1
SlitFAS2	XP_022830419.1	NvitFAS2	XP_003423914.1
SlitFAS3	XP_022832191.1	NvitFAS3	XP_001605700.1
SlitFAS4	XP_022831842.1	NvitFAS4	XP_031786889.1
SlitFAS5	XP_022831800.1	NvitFAS5	XP_008207459.3
TcasFAS1	XP_970599.2	NvitFAS6	XP_008207677.2
TcasFAS2	XP_008194480.2	NvitFAS7	XP_008212244.2
TcasFAS3	XP_015836196.1	CbowFAS1	AOA60273.1
TcasFAS4	XP_008200285.1	CbowFAS2	AMK38868.1
TcasFAS5	XP_971466.1	AgosFAS1	AKM28423.1
TcasFAS6	QBH99244.1	AgosFAS2	AKM28424.1
TcasFAS7	XP_015834319.1	AgosFAS3	AKM28425.1
TcasFAS8	XP_008190356.1	DmelFAS1	NP_608748.1
TcasFASlike1	EFA05203.2	DmelFAS2	NP_647613.1
TcasFASlike2	EFA05204.2	DmelFAS3	NP_001015405.3
TcasFASlike3	EFA11365.1	BdorFAS1	XP_011204063.1
TcasFASlike4	EFA05248.1	BdorFAS2	XP_011203074.1
TcasFASlike5	EFA05201.1	BdorFAS3	JAC37527.1

Table S3 Primers used for real-time qRT-PCR amplification of FASs

Gene name	Forward primer 5'→3'	Reverse primer 5'→3'	Product size
MpulFAS1	CATCTGTATCGTGTGGTCGTG	TTAGACCAACGTCGCCAATAG	147
MpulFAS2	CTTGGATCTGGTCGTGAATGT	TCAAACCATTGGAACGAACTC	150
MpulFAS3	GTCAATCCGTTGTCTTGCTGT	TCCACAGGTGCATTAAACACA	150
MpulFAS4	AAGAAATGAAGGCGTGGCTAT	CATTCGAGTGATCGGAGACAT	151
beta-actin	ACCTGAAGAACATCCCGTCCTTT	ACGACCAGAGGCATAAAGGGAAA	148

Table S4 Primers used to synthesize dsRNA

Primer Name	Sequence (5'-3')
MpulFAS1-Oligo-1	GATCACTAATACGACTCACTATAGGGGCGTAAATTTGGTCCTAAATT
MpulFAS1-Oligo-2	AATTTAGGACCAAATTTACGCCCCTATAGTGAGTCGTATTAGTGATC
MpulFAS1-Oligo-3	AAGCGTAAATTTGGTCCTAAACCCTATAGTGAGTCGTATTAGTGATC
MpulFAS1-Oligo-4	GATCACTAATACGACTCACTATAGGGTTTAGGACCAAATTTACGCTT
MpulFAS2-Oligo-1	GATCACTAATACGACTCACTATAGGGGCGCATTTATGGAACAATATT
MpulFAS2-Oligo-2	AATATTGTTCCATAAATGCGCCCCTATAGTGAGTCGTATTAGTGATC
MpulFAS2-Oligo-3	AAGCGCATTTATGGAACAATACCCTATAGTGAGTCGTATTAGTGATC
MpulFAS2-Oligo-4	GATCACTAATACGACTCACTATAGGGTATTGTTCCATAAATGCGCTT
MpulFAS3-Oligo-1	GATCACTAATACGACTCACTATAGGGGGGCCTTATTTCATCTCAATT
MpulFAS3-Oligo-2	AATTGAGATGAAATAAGGCCCCCCTATAGTGAGTCGTATTAGTGATC
MpulFAS3-Oligo-3	AAGGGCCTTATTTCATCTCAACCCTATAGTGAGTCGTATTAGTGATC
MpulFAS3-Oligo-4	GATCACTAATACGACTCACTATAGGGTTGAGATGAAATAAGGCCCTT
MpulFAS4-Oligo-1	GATCACTAATACGACTCACTATAGGGGGAAGCAGCTACTAAAGAATT
MpulFAS4-Oligo-2	AATTCTTTAGTAGCTGCTTCCCCCTATAGTGAGTCGTATTAGTGATC
MpulFAS4-Oligo-3	AAGGAAGCAGCTACTAAAGAACCCTATAGTGAGTCGTATTAGTGATC
MpulFAS4-Oligo-4	GATCACTAATACGACTCACTATAGGGTTCTTTAGTAGCTGCTTCCTT
GFP-Oligo-1	GATCACTAATACGACTCACTATAGGGGGGATGTCTCACATCTTGTTT
GFP-Oligo-2	AAACAAGATGTGAGACATCCCCCCTATAGTGAGTCGTATTAGTGATC
GFP-Oligo-3	AAGGGATGTCTCACATCTTGTCCCTATAGTGAGTCGTATTAGTGATC
GFP-Oligo-4	GATCACTAATACGACTCACTATAGGGACAAGATGTGAGACATCCCTT
